# The complete chloroplast genome sequence of *Malus sieboldii* (Rosaceae) and its phylogenetic analysis

**DOI:** 10.1080/23802359.2020.1768940

**Published:** 2020-05-27

**Authors:** Gongli Lou, Shuai Wang, Bin Zhang, Yueqin Cheng, Hongwei Wang

**Affiliations:** a College of Plant Protection, Henan Agricultural University, Zhengzhou, China; b College of Life Science, Henan Agricultural University, Zhengzhou, China

**Keywords:** *Malus sieboldii*, chloroplast genome, phylogenetic analysis

## Abstract

*Malus sieboldii* belongs to the genus *Malus* (Rosaceae) and is commonly considered a good rootstock for apple trees. The chloroplast (cp) genome of *M. sieboldii* was assembled using Illumina HiSeq sequencing. The whole cp genome is 160,168 bp in size, with a typical quadripartite structure consisting of a pair of inverted repeats (IR 52,716 bp), a large single-copy region (LSC 88,267 bp), and a small single-copy region (SSC 19,185 bp). The plastid genome contains 129 genes, 84 protein-coding genes, 37 tRNA genes, and 8 rRNA genes. Bayesian phylogenetic analysis showed that *M. sieboldii* is most closely related to *M. hupehensis*. The cp genome will provide more information about the taxonomy and cp evolution of the genus *Malus*.

*Malus sieboldii* (Regel) Rehd. is a perennial woody plant of the genus *Malus* (Rosaceae) (Ku and Spongberg 2003). It exhibits excellent characteristics such as strong tolerance to waterlogging, a high seed emergence rate, moderate disease resistance and drought resistance, making it an important apple rootstock resource (Sun et al. 2013). In addition, *M. sieboldii* is of great ornamental value because of its beautiful flowers in spring and colorful leaves in autumn (Walibai et al. 2017). However, the phylogenetic relationship between this species and other closely related species of the genus *Malus* has been ambiguous (Savelyeva et al. 2017). In this study, the complete chloroplast (cp) genome of *M. sieboldii* was assembled with high-throughput sequencing data to investigate its systematic position in the genus *Malus*.

A leaf sample from *M. sieboldii* was collected from Chenshan Botanical Garden (31°4′40″N, 121°10′40″E, alt. 8 m), Shanghai City, China. A specimen was deposited in the Herbarium of Henan Agricultural University (WS20190925). Total genomic DNA was extracted using the E.Z.N.A.^®^ HP Plant DNA Kit (OMEGA) (Abdulamir et al. 2010). An Illumina PE library was constructed via the genomic sequencing of DNA samples with second-generation Illumina HiSeq sequencing technology. A total of 5.9 Gb of data was obtained from the sample. The raw reads were filtered for low-quality bases (PHRED < 20) by Skewer-0.2.2 (Jiang et al. 2014). Then, using the plastid genome of *M. sieversii* (MK434920) as a reference, the clean reads were used to assemble the complete chloroplast genome. The mapped reads were extracted and assembled with NOVOPlasty-v2.7.2 (Dierckxsens et al. 2017). Then, the genome was annotated and corrected using Geneious. Finally, the circular chloroplast genome map was completed using the online program OGDRAW (Lohse et al. 2013). The whole cp genome was submitted to GenBank (accession number: MT268884).

The *M*. *sieboldii* cp genome is 160,065 bp in size, containing a large single-copy (LSC) region of 88,267 bp, a small single-copy (SSC) region of 19,185 bp, and two inverted repeat (IR) regions of 52,716 bp. The new sequence contains 129 genes, including 84 protein-coding genes, 37 tRNA genes, and 8 rRNA genes. The overall GC content of the whole plastome is 36.54%, whereas the corresponding values for the LSC, SSC, and IR regions are 34.18%, 30.39%, and 42.70%, respectively.

Here, the complete chloroplast genomes of 11 *Malus* species and *Cydonia oblonga* were aligned by using MAFFT in Geneious V.9.1 (Kearse et al. 2012). The phylogenetic tree was reconstructed by the Bayesian analysis (BI) method using chloroplast genome sequences (Ronquist et al. 2012). The results showed that *M*. *sieboldii* was closely related to *M. hupehensis* (Figure 1). This study provides a complete chloroplast genome of *M*. *sieboldii*, which can be used to analyze the polymorphism and evolution of the chloroplast genome of the genus *Malus* in future studies.

**Figure 1.**
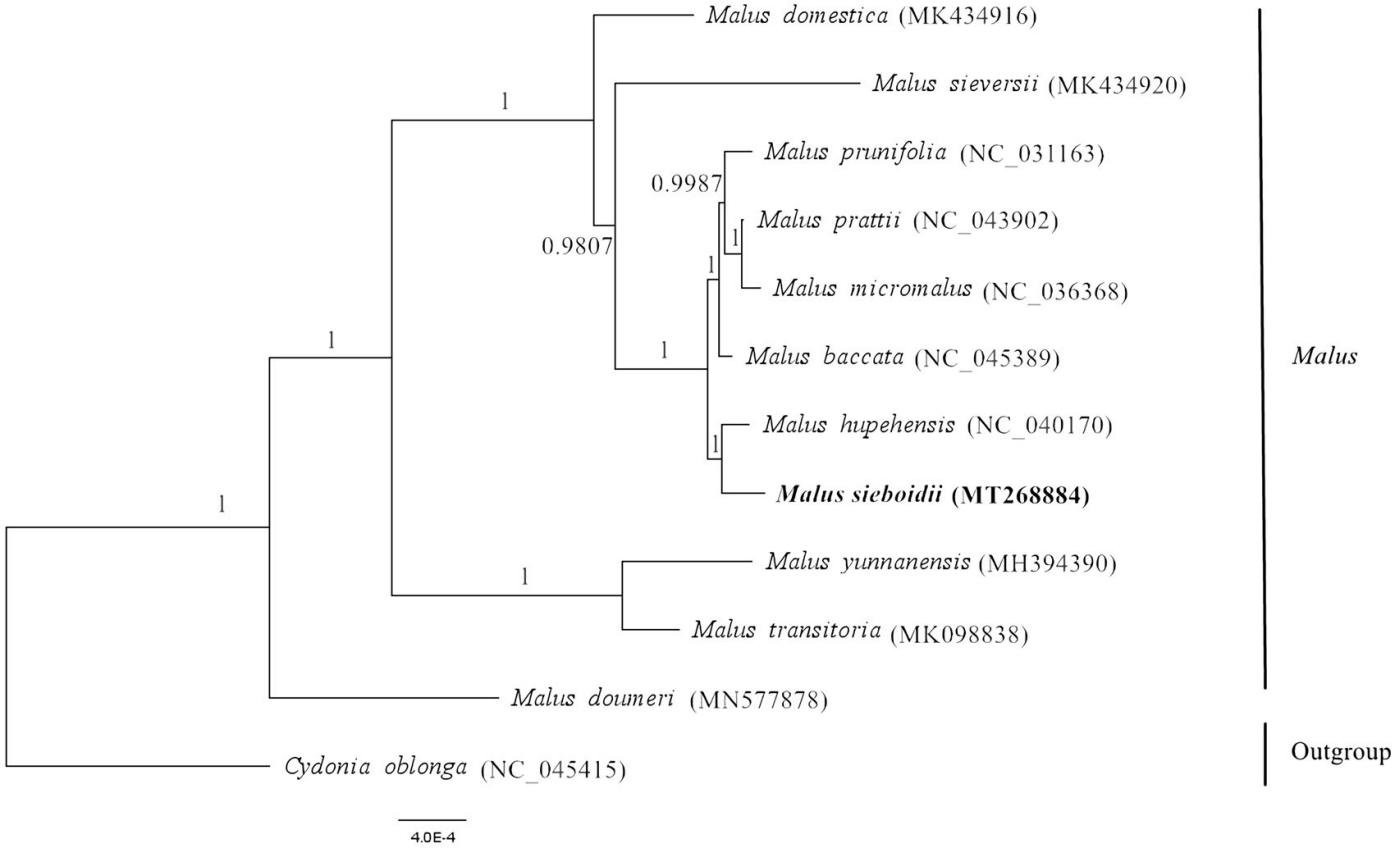
The phylogenetic tree based on the complete chloroplast genomes of 12 species. The numbers above or under the branches show the posterior probabilities. The new complete chloroplast genomes obtained in this study are shown in bold.

The data that support the findings of this study are openly available in the National Center for Biotechnology Information (NCBI) at https://www.ncbi.nlm.nih.gov/, reference number MT268884.
